# Whistle from Afar: A Case of Endotracheal Metastasis in Papillary Thyroid Cancer

**DOI:** 10.1155/2012/235062

**Published:** 2012-10-10

**Authors:** Bitoti Chattopadhyay, Biswamit Bhattacharya, Atri Chatterjee, Pijush Kanti Biswas, Nirod Baran Debnath

**Affiliations:** ^1^Department of Medicine, Nil Ratan Sircar Medical College, 138 A.J.C. Bose Road, West Bengal, Kolkata 700014, India; ^2^Department of Radiotherapy, Calcutta Medical College, Kolkata 700073, India

## Abstract

Endotracheal metastasis is a rare situation, usually associated with malignancies of breast and gastrointestinal tract, specially colon. Papillary carcinoma of thyroid commonly disseminates through lymphatic channels and tracheal involvement through vascular route is rarely reported. Here, we report a case of tracheal metastasis from papillary carcinoma of thyroid. The patient responded to external beam radiation therapy with cobalt 60 beams in a dose of 44 Gy followed by a 16 Gy boost. The patient is under followup and is presently asymptomatic. This paper adds to the repertoire of evidence in treatment of endotracheal metastasis.

## 1. Introduction

Papillary thyroid carcinoma is known for their indolent nature and erratic behavior. The disease commonly spreads to lymph nodes, often present at the time of diagnosis [1]. Though distant metastasis is uncommon, when present, it usually involves sites like lungs and bones [2]. Involvement of other structures in neck like larynx, trachea, and esophagus is usually due to direct infiltration of tumor into these structures. 

It is a notable fact that a patient may present with symptoms of upper airway obstruction in absence of any obvious thyroid swelling or lymph node enlargement. Such a situation requires differentiation from lung carcinoma, as there are significant clinicoradiological similarities [3].

## 2. Case Report 

A 40-year-old man was admitted under our care with stridor for five days. He was in his usual health till 5 months prior to admission, when he had an episode of hemoptysis. Since then, he had recurrent episodes of hemoptysis including two instances of major bleeding, which required emergency room visits. The patient was a smoker (25 pack years) and had contact history of tuberculosis. However, he did not complain of cough, shortness of breath, or chest pain. On both occasions, he had mild anemia without any other abnormality on investigation. The bleeding stopped within 24 hours of admission and the patient left hospital on risk bond.

On admission, the patient was orthopneic, the pulse was 92 beats/minute, the blood pressure was 116/72 mmHg, respiratory rate was 32/minute, and oxygen saturation was 92% while on 3 litres/minute oxygen through a nasal cannula. Apart from mild pallor, general survey was normal. There was no cervical lymphadenopathy or goiter. Examination of other systems was noncontributory. Initial investigations revealed microcytic, hypochromic anemia and normal leucocyte counts, differential, arterial blood gases, electrolytes, liver and renal function tests. Chest X-ray and ultrasonography of abdomen were normal.

**Figure 1:**
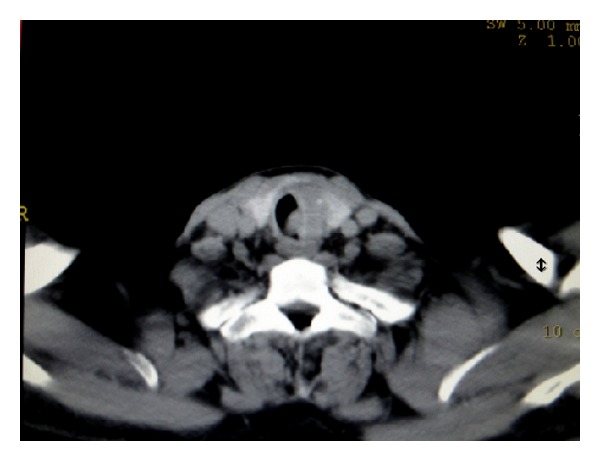
Contrast enhanced CT scan showing soft tissue mass at posterolateral wall of upper trachea (arrowhead).

**Figure 2:**
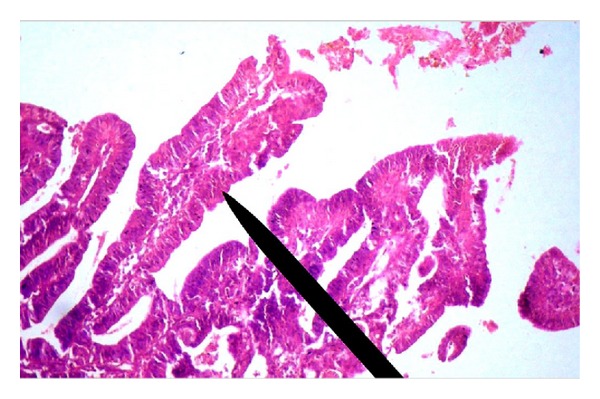
Photomicrograph (400x, H&E stain) of bronchoscopic biopsy showing papillary configuration lined by malignant cells along with prominent fibrovascular core (pointer).

On the second hospital day, the patient's symptoms worsened and computed tomography (CT) of the thorax showed a contrast-enhancing soft tissue mass in the posterolateral upper trachea to the left of the midline with normal lung parenchyma (Figure 1). On fiber-optic bronchoscopy a polypoid mass with increased vascularity was present in the upper trachea. Pathological examination of biopsy specimen from the tracheal lesion showed papillary carcinoma of the thyroid (Figure 2) and it positively stained with cytokeratin, thyroid transcription factor-1 (TTF-1), and thyroglobulin. Ki-67 labeling index was less than 1%.

**Figure 3:**
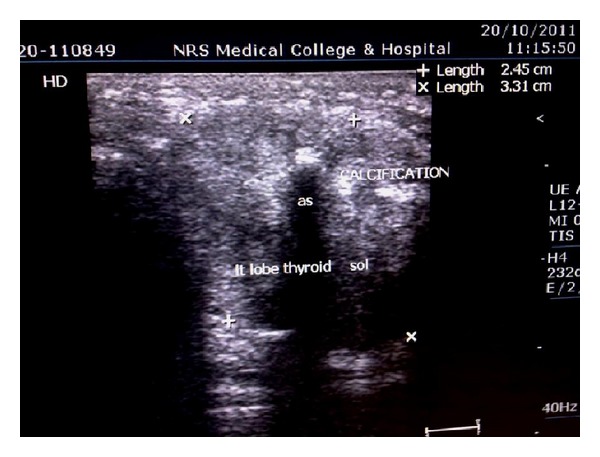
Ultrasonogram of thyroid showing calcifications with hypoechoic lesion (label).

Ultrasonography of the thyroid gland revealed two hypoechoic nodules with irregular margin with foci of microcalcification (Figure 3), the larger one measuring 1.6 cm × 1.2 cm. Ultrasound guided fine needle aspiration showed papillary carcinoma of thyroid. 

The patient did not consent to radical surgery which would necessitate a permanent tracheostomy. As an alternative, he was treated with external beam radiotherapy (EBRT) with cobalt 60 teletherapy to whole neck up to 44 Gy in 22 fractions over four and a half weeks using parallel opposed portals. After completion, a further 16 Gy boost was given to a smaller volume that included proximal trachea, whole thyroid gland, and levels II, III, and IV cervical lymph nodes.

Response to treatment was assessed with repeat CT of neck and thorax, along with bronchoscopy. The patient had complete response as per response evaluation criteria in solid tumors (RECIST), version 1.1. Presently, the patient is on follow up. He is asymptomatic and has a normal performance status for last 24 weeks.

## 3. Discussion

Tracheal metastases from a distant primary malignancy, although rare, have been documented since 1890 [4]. The most common primary cancers associated with such metastases are breast and colon, although other tumors have also been described. Thyroid malignancy with endotracheal spread has rarely been reported in literature [5]. Patterns of tracheal metastases have been classified by Kiryu et al. [6]. In the present case, the soft tissue mass arising from tracheal wall was not in continuity with thyroid nodule in imaging studies, although it yielded a diagnosis of papillary carcinoma of thyroid. It prompts us to reason that this discrete tracheal mass is a result of vascular spread, lymphatic spread being unlikely in view of absence of lymph nodal enlargement. 

Our patient belonged to type 1 according to this classification, which predicted a mean survival time of approximately 14 months. 

There are no established criteria for surgical intervention in patients with tracheal metastasis. Among other therapeutic options, external beam radiation results in acceptable survival [7]. Palliative treatment of these patients may require stenting, cryotherapy, or brachytherapy.

In conclusion, we report a case of papillary thyroid carcinoma with endotracheal metastasis, where the patient has responded to EBRT. We suggest further evaluation of EBRT as a curative therapy vis-a-vis other modalities.
